# IL-4 increases type 2, but not type 1, cytokine production in CD8^+ ^T cells from mild atopic asthmatics

**DOI:** 10.1186/1465-9921-6-67

**Published:** 2005-07-07

**Authors:** Luminita A Stanciu, Kevan Roberts, Nikolaos G Papadopoulos, Sang-Heon Cho, Stephen T Holgate, Anthony J Coyle, Sebastian L Johnston

**Affiliations:** 1 Department of Respiratory Medicine, National Heart and Lung Institute & Wright Fleming Institute for Infection and Immunity, Imperial College London, Norfolk Place, London, UK; 2 Respiratory Cell and Molecular Biology Research Division, University of Southampton, Southampton, UK; 3 MedImmune, Gaithersburg, USA

## Abstract

**Background:**

Virus infections are the major cause of asthma exacerbations. CD8^+ ^T cells have an important role in antiviral immune responses and animal studies suggest a role for CD8^+ ^T cells in the pathogenesis of virus-induced asthma exacerbations. We have previously shown that the presence of IL-4 during stimulation increases the frequency of IL-5-positive cells and CD30 surface staining in CD8^+ ^T cells from healthy, normal subjects. In this study, we investigated whether excess IL-4 during repeated TCR/CD3 stimulation of CD8^+ ^T cells from atopic asthmatic subjects alters the balance of type 1/type 2 cytokine production in favour of the latter.

**Methods:**

Peripheral blood CD8^+ ^T cells from mild atopic asthmatic subjects were stimulated *in vitro *with anti-CD3 and IL-2 ± excess IL-4 and the expression of activation and adhesion molecules and type 1 and type 2 cytokine production were assessed.

**Results:**

Surface expression of very late antigen-4 [VLA-4] and LFA-1 was decreased and the production of the type 2 cytokines IL-5 and IL-13 was augmented by the presence of IL-4 during stimulation of CD8^+ ^T cells from mild atopic asthmatics.

**Conclusion:**

These data suggest that during a respiratory virus infection activated CD8^+ ^T cells from asthmatic subjects may produce excess type 2 cytokines and may contribute to asthma exacerbation by augmenting allergic inflammation.

## Background

Asthmatic airways are characterised by high levels of IL-4 due to excess Th2 responses to common aeroallergens [1-3]. Respiratory viruses are associated with 80 to 85% of asthma exacerbations in children and with 60% in adults and rhinovirus (RV) is common in all age groups [4-6]. We have found that atopic asthmatic subjects suffer from more severe and longer lasting lower respiratory tract symptoms when infected with RV than do normal subjects [7], but the mechanisms are not known. We proposed that one mechanism could be that immune responses to virus infection are type 1 deficient/type 2 augmented if they take place in a milieu rich in the type 2 cytokine IL-4 as occurs in atopic asthma [8]. Optimal CD8^+ ^T cell responses to respiratory viruses are predominantly type 1 and over exuberant type 2 responses are detrimental [9]. Animal studies suggest that immune responses to virus infections are characterized by an increase in the frequency of type 2 cytokine-producing T cells if they take place in an allergic environment [10,11]. Data from human studies also show that an excessive type 2/deficient type 1 cytokine response to RSV-infection in the respiratory tract is associated with a more severe clinical course [12].

We have published data showing that human CD8^+ ^T cells from both normal and asthmatic subjects have the capacity to produce type 2 cytokines [13,14]. We have recently shown that stimulation of CD8^+ ^T cells from normal, healthy subjects in an IL-4 rich milieu significantly increased the number of IL-5-positive CD8^+ ^T cells [15]. Due to excess Th2 immune response to allergens, CD8^+ ^T cells from atopic asthmatic subjects are continuous exposed to IL-4 [1]. Here we investigated whether exogenous IL-4 present during TCR/CD3 stimulation modulates cell growth, surface phenotype (activation markers and adhesion molecules) and type 1 (IL-2 and IFN-γ) and type 2 (IL-4, -5, -13) cytokine production in CD8^+ ^T cells from atopic asthmatic subjects. We found that the presence of exogenous IL-4 during the activation of mild atopic asthmatic CD8^+ ^T cells enhanced cell growth and increased IL-5 and IL-13 production.

## Methods

### Subjects

Peripheral blood (40 to 50 mL) was obtained from 10 patients fulfilling clinical diagnostic criteria for asthma [16] and showing hyperreactivity to inhaled methacholine (Table 1). Asthmatic patients were classified as having mild-to-moderate asthma according to the standards of American Thoracic Society [16] and were treated with selective beta2-agonists as required. All asthmatic patients were atopic with high levels of total serum IgE (>80 IU/mL) and with positive skin prick test responses (greater than 3 mm skin wheal response) to one or more of a series of common allergens: house dust mite, mixed grass pollens, mixed tree pollens, mixed feathers, cat fur and dog hair (ALK, Denmark). None of the subjects were smokers or had a history of respiratory tract infections within the previous two months. The study was approved by Southampton Joint University and Hospital Ethics Committee.

**Table 1:** Characteristics of subjects

	Asthma subjects (*n *= 10)
Age (years)	
Mean (Range)	33 (20–48)
Sex (male/female)	5/5
PC20 methacholine (mg/mL) *	
Geometric mean (range)	5.33 (1.5 to 7.9)
Atopy^#^	10
Serum IgE (IU/mL)	
Median (range)	100 (81 to 442)

*Provocative concentration of methacholine required to cause a 20% decrease in baseline forced expiratory volume in 1 second (FEV_1_).

^# ^Positive skin reactivity to a panel of common allergen (see methods)

### Antibodies and other reagents

"CD8^+ ^T cell isolation" kits were from Miltenyi (Miltenyi Biotec GmbH, Germany). Mouse anti-human CD3 Abs was purified from the OKT-3 hybridoma (American Type Culture Collection, Rockville, USA). rIL-2 and rIL-4 were purchased from Genzyme (Genzyme, West Malling, Kent, UK). Leu 4 (CD3)-Peridinin Chlorophyll Protein conjugate, Leu 3a (CD4)-FITC and -phycoerythrin (PE), Leu 2a (CD8)-FITC and -PE, CD14-PE, CD16-PE, CD19-PE, CD25-FITC, CD11a (LFA-1)-FITC and CD49d (VLA-4)-PE were from Becton Dickinson (Becton Dickinson, Mountain View, CA). CD54 (ICAM-1)-FITC was from Serotec (Serotec Ltd, Oxford, UK). CD30-FITC and CD154 (CD40 ligand, CD40L)-PE were from PharMingen (PharMingen, San Diego, CA). ELISA kits were obtained from Biosource (Biosource Europe S.A., Fleurus, Belgium).

### CD8^+ ^T cell purification and culture

CD8^+ ^T cells were enriched by negative immunomagnetic selection using the MACS system [17]. After passage through MACS columns, between 0.25 and 2.3 × 10^6 ^CD8^+ ^T cells were recovered from 1 mL of blood. The resulting cell populations were routinely >98% CD3^+ ^and >97% CD8^+ ^and contained no detectable CD4^+^, CD16^+^, CD19^+^, CD14^+ ^cells as determined by flow cytometry.

Purified CD8^+ ^T cells were cultured in an antigen presenting cell-free system as previously described [15]. In brief, 0.5 × 10^6 ^cells/mL were stimulated for 3 days with plate-bound anti-CD3 Abs (10 μg/mL) in 96-well microtiter plates in RPMI 1640 culture medium supplemented with 10% FCS, 2 mM L-glutamine, 1 mM sodium pyruvate, antibiotics (all from Gibco BRL, Life Technologies, Uxbridge, UK), IL-2 (50 U/mL) with or without added IL-4 (10 ng/mL) (stimulation). After 3 days, the cells were harvested, washed, adjusted to 0.2 × 10^6^/mL and cultured for 4 days in culture medium containing IL-2 (50 U/mL) (expansion). After every cycle of stimulation or expansion the cells were harvested and the number of live cells was determined by trypan blue exclusion and counting. After the second cycle of stimulation and expansion the cells were harvested, washed, adjusted to 1 × 10^6^/mL and analysed.

### Cell surface markers

Following the second cycle of stimulation/expansion, the cells were harvested and washed. PerCP-, FITC-, and PE-conjugated antibodies were added to 50 μl of suspensions (0.05 × 10^6 ^cells) and incubated for 30 min at 4°C. After washing in PBS, stained cells were resuspended in 500 μl PBS. Flow cytometry was performed on a FACScan (Becton Dickinson, San Jose, CA). The FACScan program was used to acquire data of 10,000 events and the data were analysed by using Lysys II software. Live lymphocytes were gated by the forward scatter and side scatter pattern. Percentage in dot plots and mean fluorescence intensity (MFI) in histograms were analysed on CD3^+^CD8^+ ^cells for the molecules of interest.

### Cytokine production

After two cycles of stimulation/expansion, CD8^+ ^T cells (1 × 10^6^/mL) were restimulated for 24 hours with phorbol myristate acetate (PMA) (20 ng/mL) and ionomycin (2 μg/mL) or with immobilised anti-CD3 Ab. Supernatants were microfuged for 5 minutes at 400 g to remove cell debris and IL-2, IL-4, IL-5, IL-13, and IFN-γ levels were measured by ELISA using commercial assay kits (Biosource, Europe SA). The limits of sensitivity of these assays were 3 pg/mL for IL-4, 4 pg/mL for IL-5 and IFN-γ, 5 pg/mL for IL-2, and 12 pg/mL for IL-13.

### Statistical analysis

Paired Student t-test was used to analyse normally distributed data such as growth of CD8^+ ^T cells cultured with or without IL-4. The Wilcoxon signed rank test for paired data was used for comparison of surface markers and for cytokine levels in culture supernatants. *P *values less than 0.05 were chosen for rejection of the null hypothesis.

## Results

### IL-4 amplifies the growth of CD8^+ ^T cells from atopic asthmatic subjects

**Figure 1:**
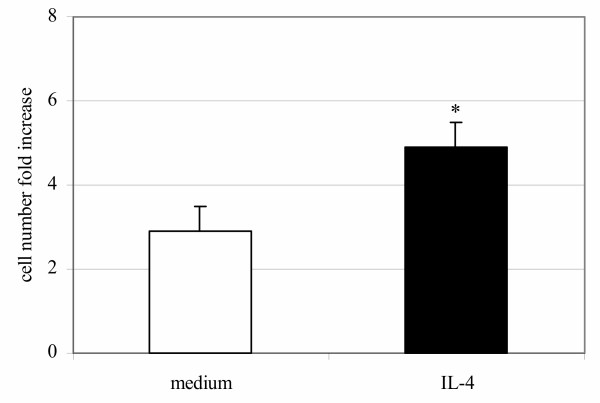
**Growth kinetics (fold increase) of CD8^+ ^T cell numbers**. Data are means ± SEM (n = 10 subjects). * p < 0.02

In order to analyse the effect of IL-4 on CD8^+ ^T cell growth we examined cell recovery after the first and second cycles of stimulation and expansion. At the end of the first cycle of stimulation/expansion there was no difference in the number of CD8^+ ^T cells cultured with or without IL-4 (data not shown), but after the second cycle the number of cells stimulated in the presence of IL-4 was significantly higher than number of cells stimulated in the absence of IL-4 (4.5 ± 0.7 fold increase relative to the initial population in the presence of exogenous IL-4 versus 2.9 ± 0.7 fold increase without exogenous IL-4 (Fig. 1, *P *< 0.02).

### IL-4 decreases surface expression of adhesion molecules in CD8^+ ^T cells from atopic asthmatic subjects

As we were interested in airway responses of CD8^+ ^T in the context of viral infection, we then investigated whether levels of expression of surface antigens associated with activation, adhesion and transmigration capabilities were altered by the presence of IL-4 during CD8^+ ^T cell stimulation (Table 2). There was a trend to increased numbers of CD30-positive CD8^+ ^T cells in culture with exogenous IL-4 but the difference did not reach significance (*P *< 0.07). However, the presence of exogenous IL-4 during stimulation induced a significant decrease in the expression (assessed by MFI) on CD8^+ ^T cells of the adhesion molecules VLA-4 (CD29/CD49d) (*P *< 0.02) and LFA-1 (*P *< 0.05). There was a trend towards a similar reduction in ICAM-1 expression, though this did not reach statistical significance (*P *< 0.06).

**Table 2:** Surface molecules on repeatedly stimulated CD8^+ ^T cells*

IL-4	-	+	*P*^†^
CD3^+^	97 ± 1	98 ± 0.5	NS
CD3^+^CD8^+^CD4^-^	97 ± 1	97 ± 1	NS
CD3^+^CD4^+^CD8^-^	0.2 ± 0.2	0.3 ± 0.2	NS
CD3^+^CD4^-^CD8^-^	2 ± 1	2 ± 1	NS
CD3^+^CD4^+^CD8^+^	7 ± 6	4 ± 1	NS
			
CD3^+^CD8^+^			
CD25	53 ± 9	68 ± 9	NS
CD30	23 ± 7	40 ± 6	0.07
CD40L	14.5 ± 5	14.5 ± 6	NS
VLA-4^#^	444 ± 97	283 ± 72	0.02
LFA-1^#^	280 ± 51	194 ± 25	0.05
ICAM-1^#^	37 ± 10	27 ± 7	0.06

*frequency of positive cells or ^#^mean fluorescence intensity of surface antigens after two rounds of stimulation of CD8^+ ^T cells from atopic asthmatic subjects (mean ± SEM; *n *= 7; NS, nonsignificant).

^† ^comparison between IL-4-treated and -untreated cells (paired Student's t test)

### IL-4 enhances type 2 cytokine production by CD8^+ ^T cells from atopic asthmatic subjects

**Figure 2:**
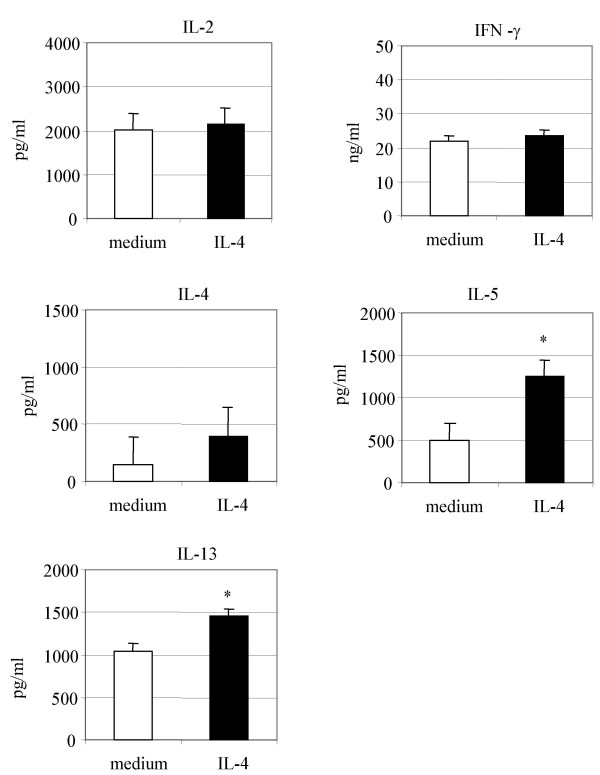
**Exogenous IL-4 during TCR/CD3 stimulation increased type 2 cytokine production in culture supernatants of CD8^+ ^T cells from atopic asthmatic subjects**. CD8^+ ^T cells were stimulated with anti-CD3 Abs+IL-2 in the presence or absence of IL-4 for 3 days followed by 4 days expansion with IL-2 alone- this cycle of stimulation/expansion was then repeated. Cells were then harvested and restimulated (1 × 10^6^/mL) with PMA and ionomycin. Supernatants were collected after 24 hours, and cytokines measured by ELISA. Cytokine data are median (quartiles 75) of 10 atopic asthmatic subjects. * p < 0.05

To investigate whether the presence of IL-4 during CD8^+ ^T cell stimulation alters the cytokine production pattern, CD8^+ ^T cells harvested after two cycles of stimulation/expansion were restimulated with PMA/ionomycin or immobilized anti-CD3 Abs for 24 h to investigate cytokine synthesis and release into supenatants. The patterns of alteration in cytokine secretion following 24 h restimulation with anti-CD3 Abs (data not shown) were similar to that observed after PMA and ionomycin restimulation (Fig. 2).

Type 2 cytokine production by repeatedly stimulated CD8^+ ^T cells from atopic asthmatic subjects was increased by the presence of IL-4 during stimulation (Fig. 2). There was a trend towards an increase in IL-4 (median and [range]) production by CD8^+ ^T cells stimulated in the presence of IL-4 (without IL-4, 146 pg/mL [20 to 793 pg/mL]; with IL-4, 389 pg/mL [45 to 4309 pg/mL], *P *< 0.1). IL-5 levels were significantly increased by the presence of IL-4 (without IL-4, 494 pg/mL [56 to 1612 pg/mL]; with IL-4, 1251 pg/mL [391 to 10936 pg/mL], *P *< 0.02). IL-13 levels were also significantly increased by the presence of IL-4 during CD8^+ ^T cell stimulation (without IL-4, 1045 pg/mL [378 to 1392 pg/mL]; with IL-4, 1455 pg/mL [580 to 5820 pg/mL], *P *< 0.01).

Excess IL-4 during repeated stimulation of CD8^+ ^T cells did not significantly alter the levels of IL-2 (without IL-4, 2000 pg/mL [1000 to 4000 pg/mL]; with IL-4, 2135 pg/mL [1000 to 2900 pg/mL]) and IFN-γ (without IL-4, 22 ng/mL [2 to 44 ng/mL]; with IL-4, 23 ng/mL [1 to 44 ng/mL] released in culture by CD8^+ ^T cells.

## Discussion

In this study, we show that excess exogenous IL-4 during *in vitro *CD8^+ ^T cell stimulation amplified the proliferation of CD8^+ ^T cells from atopic asthmatic subjects. Furthermore, excess IL-4 resulted in a decrease in the expression of the adhesion molecules VLA-4 and LFA-1 on CD8^+ ^T cells, along with increased production of the type 2 cytokines IL-5 and IL-13. These data indicate that the activation of CD8^+ ^T cells from asthmatic subjects in the presence of IL-4 increases their capacity to produce type 2 cytokines but not IFN-γ or IL-2. These effects may be important in promoting adverse CD8^+ ^T cell responses to virus infections in asthmatic subjects.

The presence of exogenous IL-4 during TCR/CD3 stimulation of peripheral blood CD8^+ ^T cells induced expansion of cell numbers. It has previously been reported that IL-4 may act as a growth factor for human CD4^+ ^and CD8^+ ^T cell clones and that there is a synergy between IL-2 and IL-4 during CD8^+ ^T cell proliferation [18]. This suggests that stimulation of CD8^+ ^T cells in an environment rich in IL-4 may result in the expansion of CD8^+ ^T cells when exogenous IL-2 is present.

CD8^+ ^T cells exposed to repeated stimulation in the presence of exogenous IL-4 decreased their expression of the α_4_β_1 _integrin (VLA-4, CD49d/CD29) and α_L_β_2 _integrin (LFA-1, CD11a/CD18). VLA-4 is a surface marker associated with the capacity of T cells to migrate towards sites of antigen stimulation [19] and it has been previously reported that CD49d membrane expression on Th1 and Th2 clones is markedly reduced by activation [20]. The reduction in the levels of adhesion molecules as a result of activation of CD8^+ ^T cells may play a role in the retention of activated cells at sites of antigenic stimulation. Recently, in an animal model it was reported that type 2 or type 0 CD8^+ ^T cells had decreased surface VLA-4 density and deficient activation-induced LFA-1/ICAM-1-dependent homotypic adhesion *in vitro *and consequently reduced antiviral activity *in vivo *[21]. The presence of exogenous IL-4 during stimulation of CD8^+ ^T cells also tended to increase the frequencies of CD30-positive cells. The expression of CD30, a member of the TNF receptor family, by T cells has been previously reported to be primarily regulated by IL-4 [22] and preferentially expressed by type 2 and type 0 cells [20]. These data collectively suggest that the presence of IL-4 in the microenvironment induces a phenotype of cells similar to tissue-resident cells and induces patterns of expression of adhesion molecules associated with adoption of type 2 function. These data suggest our *in vitro *observations on induction of type 2 functions more likely to truly reflect events occurring in the lungs *in vivo*.

We have found that peripheral blood CD8^+ ^T cells from atopic asthmatic individuals, when stimulated in the presence of exogenous IL-4, released higher levels of IL-5 and IL-13 as compared with CD8^+ ^T cells stimulated in the absence of IL-4. It has previously been reported that exogenous IL-4 increased production of IL-4 and IL-5 in stimulated neonatal CD8^+ ^T cells [23]. However, it is now known that neonatal T cells are skewed towards a type 2 phenotype and may, therefore, not be representative of adult human CD8^+ ^T cells [24]. We have recently reported induction of type 2 activities in CD8^+ ^T cells from healthy adult subjects during TCR stimulation by the presence of exogenous IL-4 [15]. However, we observed only a tendency to increased IL-5-production and no increased IL-13-production in CD8^+ ^T cells from normal subjects [[15] and unpublished data]. In the present study we extend these findings to confirm that induction by IL-4 of type 2 activities also occurs in CD8^+ ^T cells from asthmatic subjects and demonstrate the deviation in phenotype towards type 2 is more extensive with increases in IL-5 and IL-13 production.

Respiratory virus infections are frequently associated with asthma exacerbations and CD8^+ ^T cells are important in anti-viral immune responses. Given the above data in our *in vitro *model, we suggest that stimulation of CD8^+ ^T cells during viral infections in an environment enriched in IL-4 as occurs in asthma is likely to favour the induction of type 2 cytokine synthesis by CD8^+ ^T cells. Production of type 2 cytokines by CD8^+ ^T cells during immune responses to viral infections in asthma has the potential to augment allergic inflammation by a number of different mechanisms including each of the type 2 cytokines measured in this study. This hypothesis is lent credence by our recent observations that virus infection and allergen exposure are synergistic risk factors for acute exacerbations of asthma [25].

By antagonising the production of IFN-γ, type 2 cytokines may also have effects on the capacity of CD8^+ ^T cells to clear virus. In our culture conditions, even though exogenous IL-4 did not significantly decrease the levels of IFN-γ, the levels remained stable and the ratio of IL-4/IFN-γ was increased (data not shown). It is quite possible that during respiratory viral infections in asthmatic relative to normal subjects *in vivo*, where the stimulation conditions are different, IFN-γ production is depressed as a result of these mechanisms. This hypothesis is supported by the observation in mice that elevation of IL-4 levels concurrent with viral infections suppresses or delays activation of virus-specific CD8^+ ^T cells and leads to delayed viral clearance [26-28]. We have previously reported a significant increase in submucosal CD4^+ ^and CD8^+ ^lymphocytes and in epithelial eosinophils in both normal and asthmatic subjects with experimental rhinovirus infections, and in asthmatic subjects eosinophil numbers remained elevated during convalescence [29]. These data suggest that modulation by IL-4 of CD8^+ ^virus-specific immune responses towards a type 2 phenotype may occur in humans *in vivo*, and that this may be an important mechanism in virus-induced exacerbations of asthma acting both by augmenting allergic inflammation and by diminishing viral clearance. A recent rhinovirus experimental infection study has supported this hypothesis by observing impaired virus clearance and increased symptoms in subjects with increased IL-5/IFN-γ mRNA ratios in induced sputum [30].

## Conclusion

In conclusion, our data demonstrate induction of type 2 cytokine production and a tissue-resident phenotype by exogenous IL-4 in CD8^+ ^T cells from atopic asthmatic subjects. Given the excess IL-4 observed in asthma we believe that these mechanisms may play an important role in impairing virus clearance and augmenting allergic inflammation in virus-induced asthma. We are currently testing this possibility *in vivo *in human experimental virus infection models.

## Competing interests

The author(s) declare that they have no competing interests.

## Authors' contributions

LAS participated in the design of the study, performed the studies and drafted the manuscript. KR participated in the design of the study. NGP participated in the design of the study. SHC participated in the design of the study. STH conceived of the study. AJC conceived of the study. SLJ conceived of the study, participated in its design and coordination and helped to draft the manuscript. All authors read and approved the final manuscript.
